# Factors Associated With Daily Consumption of Sugar-Sweetened Beverages Among Adult Patients at Four Federally Qualified Health Centers, Bronx, New York, 2013

**DOI:** 10.5888/pcd12.140342

**Published:** 2015-01-08

**Authors:** Ross B. Kristal, Arthur E. Blank, Judith Wylie-Rosett, Peter A. Selwyn

**Affiliations:** Author Affiliations: Arthur E. Blank, Peter A. Selwyn, Montefiore Medical Center, Albert Einstein College of Medicine, Bronx, New York; Judith Wylie-Rosett, Albert Einstein College of Medicine, Bronx, New York.

## Abstract

**Introduction:**

Consumption of sugar-sweetened beverages (SSBs) is associated with cardiovascular disease risk factors. This study examined the relationships between SSB consumption and demographic, health behavior, health service, and health condition characteristics of adult patients of a network of federally qualified health centers (FQHCs) in a low-income, urban setting.

**Methods:**

Validated, standardized self-reported health behavior questions were incorporated into the electronic health record (EHR) and asked of patients yearly, at 4 FQHCs. We conducted cross-sectional analysis of EHR data collected in 2013 from 12,214 adult patients by using logistic regression.

**Results:**

Forty percent of adult patients consumed 1 or more SSBs daily. The adjusted odds ratios indicated that patients who consumed more than 1 SSB daily were more likely to be aged 18 to 29 years versus age 70 or older, current smokers versus never smoking, eating no servings of fruits and/or vegetables daily or 1 to 4 servings daily versus 5 or more servings daily, and not walking or biking more than 10 blocks in the past 30 days. Patients consuming 1 or more servings of SSBs daily were less likely to speak Spanish than English, be women than men, be diagnosed with type 2 diabetes versus no diabetes, and be diagnosed with hypertension versus no hypertension.

**Conclusion:**

SSB consumption differed by certain demographic characteristics, health behaviors, and health conditions. Recording SSB intake and other health behaviors data in the EHR could help clinicians in identifying and counseling patients to promote health behavior changes. Future studies should investigate how EHR data on patient health behavior can be used to improve the health of patients and communities.

## Introduction

On a given day, half of the US population consumes a sugar-sweetened beverage (SSB) (1). SSB intake is associated with weight gain, high blood pressure, type 2 diabetes, and metabolic syndrome — all of which are risk factors for cardiovascular disease, 1 of the leading causes of preventable death in the United States (2–9). People living in poverty and having food insecurity are disproportionally burdened by some of these risk factors for many reasons, including the low cost of energy-dense foods such as SSBs (10). Previous studies have examined the associations of behavioral and sociodemographic characteristics with SSB intake among adults at national, state, and regional levels (1,11–15). However, to our knowledge, no studies measure these associations among a patient population in a primary care setting. Primary care providers (PCPs) can reduce the number of preventable cardiovascular-related deaths by discussing health-related lifestyle behaviors and promoting behavior change with their patients (16). Correspondingly, primary care centers that incorporate patient-reported health behaviors such as SSB consumption into their electronic health record (EHR) systems can share this information with public health agencies and help them coordinate health promotion among patients and other populations (17,18). In addition, the data may be used to examine patient outcomes in the context of population trends (19). Better understanding the sociodemographic and behavioral factors of this high-risk population can help public health and clinical systems improve efforts to reduce SSB consumption.

The primary objective of this study was to examine the relationships between SSB consumption and demographic, health behavior, health service, and health condition characteristic variables of adult patients served by a network of hospital-affiliated federally qualified health centers (FQHCs) in a low-income urban setting.

## Methods

### Data sources

In 2010, a multistakeholder effort entitled Bronx CATCH (Collective Action to Transform Community Health), and led by Montefiore Medical Center (MMC), the New York City Department of Health and Mental Hygiene (DOHMH), and the Bronx Community Health Network, was established to improve the health of patients and communities (20). Agreeing that the primary care and public health sectors have a common goal of improving health and that community factors (eg, access to recreational areas) and clinical interventions (eg, prescribing cholesterol-lowering medication) are complementary in improving health, stakeholders decided to collaborate to achieve this common goal. In addition to implementing patient-based services such as EHR-based diabetes prevention program referrals and community-based services such as working with grocers to promote healthier food options, in 2012, Bronx CATCH incorporated 5 standardized self-reported questions about dietary intake and physical activity habits into MMC’s EHR structure. These questions were chosen from the New York City Community Health Survey (CHS), a telephone survey conducted annually by the New York City DOHMH using questions from the Centers for Disease Control and Prevention Behavioral Risk Factor Surveillance System (21,22). Two of the 5 CHS questions quantify daily SSB consumption. These questions were incorporated into the EHR for all adults and children aged 6 years or older, and programmed to appear routinely at the first visit of the calendar year for all patients and then to recur annually. This report includes data only from patients aged 18 years or older.

After integrating the 5 CHS dietary and physical activity lifestyle behavior questions into the EHR structure, responses to the questions were systematically incorporated into the workflow of select MMC-affiliated FQHCs. If adult patients did not have responses to the risk assessment questions (RAQs) documented in the EHR within the past year, then the EHR would automatically prompt the nurse to ask the questions during the vital signs assessment and input the patient’s responses. The answers, along with clinical and demographic data, were extracted from the EHR by MMC information technology personnel.

### Study population and inclusion criteria

Patients in the MMC EHR database were selected for study inclusion if the database indicated they 1) received care from 1 of the 4 FQHCs included in the study, 2) answered the SSB questions in 2013, 3) were aged 18 or older, and 4) had sufficient information in their medical record to be classified as having type 2 diabetes, prediabetes, or no diabetes. All patients who met the 4 inclusion criteria were included in the study. There was no a priori power calculation.

The outcome variable for this analysis was binary and scored 1 if an individual had less than 1 SSB a day and 0 if he or she consumed 1 or more daily servings of SSB. The number of SSB drinks was the sum of the answers to 2 questions: “How often do you drink sugar-sweetened soda? Do not include diet soda or seltzer,” and “How often do you drink other sweetened drinks like sweetened iced tea, sports drinks, fruit punch, or other fruit-flavored drinks? Do not include diet soda, sugar-free drinks, or 100% juice.” As stated in the New York City CHS, 1 SSB serving was defined as 12 ounces (22). Answers could have been given per day, per week, or per year, and all answers were standardized to number of servings per day.

### Variables

We included numerous covariates in the analysis. These covariates included demographic, health behavior, health service, and health condition variables.

The demographic variables were age, sex, race/ethnicity, and language preference. The reported age was the individual’s age, determined by the individual’s birth date when the SSB RAQs were answered. Race/ethnicity and language preference were self-reported by patients when they were first seen at the health center. Race and ethnicity were reported separately by the patient. The derived variable was a combination of the 2 responses. People who identified as Latino or Hispanic were coded as that ethnic group regardless of any additional racial classification. The racial classification was used to code the race/ethnicity variable for patients who reported themselves as non-Hispanic or non-Latino. All racial categories were mutually exclusive. Patients could have declined to answer the question.

This study included 4 health behavior variables. First, smoking status was defined as the most recent smoking status in the EHR dating 5 years before answering the SSB RAQs. Second was the number of servings of daily fruit and/or vegetable consumption. Examples of 1 serving are 1 medium apple, a handful of broccoli, or a cup of carrots. Third is participation in physical activity or exercise other than the individual’s regular job in the past 30 days, such as running, calisthenics, golf, gardening, or walking. Fourth is walking or biking more than 10 blocks as part of getting to and from work, school, public transportation or to do errands in the past 30 days. The last 3 questions were consistent with the New York City CHS (22).

The health services variables included insurance status as well as the number of outpatient visits at any of the 4 FQHCs in the last year before answering the SSB RAQs. Insurance status was coded using the payment method during the clinical visit on the day of or latest day before answering the SSB RAQs.

Weight status, diabetes status, and the diagnosis of hypertension and hyperlipidemia were categorized as health condition variables. Weight status was classified using body mass index (BMI) categories as underweight/normal weight (<25.0 kg/m^2^), overweight (25.0–29.9 kg/m^2^) or obese (≥30.0 kg/m^2^). We used the measurement closest to the date of answering the SSB RAQs that was within 14 days before or after answering the SSB RAQ. Patients with BMI values <15 kg/m^2^ and >85 kg/m^2^ were considered outliers.

We used hemoglobin A1C (HbA1C) results, International Classification of Diseases, Ninth Revision (ICD-9) codes, and a diagnosis list starting in 2010 to categorize patients as having no diabetes, prediabetes, or type 2 diabetes. The January 2010 start date was chosen because it coincided with the American Diabetes Association’s (ADA’s) update of its recommendations to categorize an HbA1C of 5.7%–6.49% as prediabetes and an HbA1C of ≥6.5% as overt diabetes (23). The scheme used to categorize diabetes status was adopted from previously published studies (24–26).

Patients were considered to have hypertension if they had an ICD-9 diagnosis of 401.1 or 401.9 and a diagnosis of hyperlipidemia if they had an ICD-9 diagnosis of 272.0–272.4. The analyses were conducted by using a parameter searching for the ICD-9 diagnosis 1 year and 5 years before answering the SSB RAQs. There were nonsignificant differences in outcomes when using either the 1-year or 5-year parameter, so only the 5-year parameter was reported.

### Statistical analysis

Pairwise deletion was used in the analysis, given the small percentage of missing, unknown and outlier values, with the exception of race/ethnicity. The race/ethnicity variable had a higher percentage of missing or unknown values, so an additional category, unknown/missing/refused, was made for these individuals and they were included in the analysis. This methodology of using pairwise deletion with the exception of variables with a high proportion of missing or unknown values, in which an additional category was created, was adopted from Park et al (13).

Bivariate analysis using Pearson χ^2^ test for categorical variables was performed to assess the association of the covariates between subjects who consumed <1 and ≥1 SSB daily. During the model building stage, crude odds ratios (ORs) and 90% confidence intervals (CIs) were calculated for the dependent variable and all covariates. Variables with significant differences (*P* < .10) and all demographic variables were entered into a logistic regression model. To test the final model, adjusted ORs and 95% CIs were determined. Demographic variables were categorically entered into the model given the known differences in health disparities for different demographic groups. All analyses were conducted in R version 3.0.1 (the R project for statistical computing).

## Results

Of the 13,072 adult patients who answered the RAQs at the 4 participating FQHCs in 2013, 858 patients were excluded from the study because they did not answer the SSB RAQs (n = 58) or were classified as having type 1 diabetes or an undetermined diabetes classification (n = 800). Missing or unknown values ranged from 0.22% for the walking or biking covariate to 4.90% for the weight status covariate. Thirty-nine percent of subjects had missing or unknown race/ethnicity classification. The sample population characteristics are reported (Table 1). The 4 participating FQHCs had 36,100, 12,102, 19,260, and 32,994 total adult patient visits in 2013. The characteristics of the community where the FQHCs are located are reported in the Appendix.

**Table 1:** Characteristics of Adult Patients at Federally Qualified Health Centers Who Answered Questions About Consumption of Sugar-Sweetened Beverages, Bronx, New York, 2013

Variable	n (%^a^)
**Demographic variables**	
**Age, y**	**12,214 (100.0)**
18–29	2,526 (20.7)
30–39	1,882 (15.4)
40–49	2,202 (18.0)
50–59	2,465 (20.2)
60–69	1,728 (14.4)
≥70	1,411 (11.6)
**Sex**	**12,214 (100.0)**
Male	3,495 (28.6)
Female	8,719 (71.4)
**Race/ethnicity**	**12,214 (100.0)**
White	58 (0.5)
Black or African American	1,259 (10.3)
Hispanic or Latino	5,924 (48.5)
Asian	56 (0.5)
Other	156 (1.3)
Unknown/missing/refused	4,761 (39.0)
**Preferred language**	**12,132 (100.0)**
English	9,224 (76.0)
Spanish	2,632 (21.7)
Vietnamese	50 (0.4)
Cambodian	43 (0.4)
Other	183 (1.5)
**Health behavior variables**	
**Smoking status**	**12,103 (100.0)**
Never smoked	7,754 (64.1)
Former smoker	2,215 (18.3)
Current smoker	2,134 (17.6)
**Fruit and/or vegetable consumption, servings/day^b^**	**12,157 (100.0)**
0	3,231 (26.6)
1–4	8,504 (70.0)
5 or more	422 (3.5)
**Exercise^c^**	**12,214 (100.0)**
Yes	8,198 (67.1)
No	4,016 (32.9)
**Walk/Bike^d^**	**12,188 (100.0)**
Yes	7,306 (60.0)
No	4,882 (40.1)
**Health service variables**	
**Insurance**	**11,816 (100.0)**
Medicare or commercial	5,067 (43.6)
Medicaid or uninsured	6,543 (56.4)
**Outpatient PCP visits per year**	**12,214 (100.0)**
1	1,712 (14.0)
2–6	4,546 (37.2)
7–12	3,221 (26.4)
≥13	2,735 (22.4)
**Health condition variables**	
**Weight status**	**11,816 (100.0)**
Underweight/normal weight (BMI <25 kg/m^2^)	2,735 (23.2)
Overweight (BMI 25–29.9 kg/m^2^)	3,851 (32.6)
Obese (BMI ≥30 kg/m^2^)	5,230 (44.3)
**Diabetes status**	**12,214 (100.0)**
No diabetes	7,107 (58.2)
Prediabetes	2,715 (22.2)
Type 2 diabetes	2,392 (19.6)
**Hypertension**	**12,214 (100.0)**
No	6,828 (55.9)
Yes	5,386 (44.1)
**Hyperlipidemia**	**12,214 (100.0)**
No	8,193 (67.1)
Yes	4,021 (32.9)

Abbreviations: BMI, body mass index; PCP, primary care provider.

a Percentages may not total 100% because of rounding.

b One serving equals 1 medium apple, a handful of broccoli, or a cup of carrots.

c Participating in any physical activities other than an individual’s regular job or exercises such as running, calisthenics, golf, gardening, or walking for exercise in the past 30 days.

d Walking or biking more than 10 blocks as part of getting to and from work, school, public transportation or to do errands in the past 30 days.

Patient characteristics by level of SSB intake are provided in Table 2. Although 40.2% of the study population consumed 1 or more servings of SSBs per day, the proportion was highest among patients aged 18 to 29, male, and preferentially English speaking. Among health behavior characteristics, patients who were current smokers, ate less than 5 servings of fruits or vegetables daily, and walked or biked less than 10 blocks in the past 30 days had the highest prevalence of consuming 1 or more SSBs per day. People with Medicaid or no health insurance and individuals with 1 PCP visit had the highest proportion of participants who drank 1 or more SSBs daily. Adults with healthier clinical measurements, including being underweight/normal weight and not having already-diagnosed diabetes, hypertension, or hyperlipidemia, had the highest prevalence of daily SSB consumption (*P* < .001 for all variables based on χ^2^ tests).

**Table 2:** Prevalence of Sugar-Sweetened Beverage Consumption and Association With Characteristics Among Adult Patients Seen at a Federally Qualified Health Center in Bronx, New York, 2013

Variable	Sugar-Sweetened Beverage Consumption^a^		
	<1 Serving/Day, n (%^b^)	≥1 Serving/Day, n (%^b^)	*P* Value^c^
**Demographic variables**			
**Age, y**	**7,303 (59.8)**	**4,911 (40.2)**	
18–29	1,137 (45.0)	1,389 (55.0)	<.001
30–39	1,029 (54.7)	853 (45.3)	
40–49	1,305 (59.3)	897 (40.7)	
50–59	1,625 (65.9)	840 (34.1)	
60–69	1,211 (70.1)	517 (29.9)	
≥70	996 (70.6)	415 (29.4)	
**Sex**	**7,303 (59.8)**	**4,911 (40.2)**	
Male	1,970 (56.4)	1,525 (43.6)	<.001
Female	5,333 (61.2)	3,386 (38.8)	
**Race/ethnicity**	**7,303 (59.8)**	**4,911 (40.2)**	
White	31 (53.4)	27 (46.6)	.48
Black or African American	752 (59.7)	507 (40.3)	
Hispanic or Latino	3,526 (59.5)	2,398 (40.5)	
Asian	40 (71.4)	16 (28.6)	
Other	93 (59.6)	63 (40.4)	
Unknown/missing/refused	2,861 (60.1)	1,900 (39.9)	
**Preferred language**	**7,251 (59.8)**	**4,881 (40.2)**	
English	5,318 (57.7)	3,906 (42.3)	<.001
Spanish	1,731 (65.8)	901 (34.2)	
Vietnamese	38 (76.0)	12 (24.0)	
Cambodian	34 (79.1)	9 (20.9)	
Other	130 (71.0)	53 (29.0)	
**Health behaviors**			
**Smoking status**	**7,246 (59.9)**	**4,857 (40.1)**	
Never smoked	4,897 (63.2)	2,857 (36.8)	<.001
Former smoker	1,379 (62.3)	836 (37.7)	
Current smoker	970 (45.5)	1,164 (54.5)	
**Fruit and/or vegetable consumption, servings/day^d^**	**7,279 (59.9)**	**4,878 (40.1)**	
None	1,649 (51.0)	1,582 (49.0)	<.001
1–4 servings	5,312 (62.5)	3,192 (37.5)	
5 or more servings	318 (75.4)	104 (24.6)	
**Exercise^e^**	**7,303 (59.8)**	**4,911 (40.2)**	
Yes	4,912 (40.2)	3,286 (40.1)	.68
No	2,391 (19.6)	1,625 (40.5)	
**Walk/bike^f^**	**7,288 (59.8)**	**4,900 (40.2)**	
Yes	4,512 (61.8)	2,794 (38.2)	<.001
No	2,776 (56.9)	2,106 (43.1)	
**Health service**			
**Insurance**	**7,288 (59.8)**	**4,900 (40.2)**	
Medicare or commercial	4,512 (61.8)	2,794 (38.2)	<.001
Medicaid or uninsured	2,776 (56.9)	2,106 (43.1)	
**Outpatient PCP visits per year**	**7,303 (59.8)**	**4,911 (40.2)**	
1	892 (52.1)	820 (47.9)	<.001
2–6	2,612 (57.5)	1,934 (42.5)	
7–12	2,028 (63.0)	1,193 (37.0)	
≥13	1,771 (64.8)	964 (35.2)	
**Health conditions**			
**Weight status**	**7,074 (59.9)**	**4,742 (40.1)**	
Underweight/normal weight (BMI <25.0 kg/m^2^)	1,517 (55.5)	1,218 (44.5)	<.001
Overweight (BMI 25.0–29.9 kg/m^2^)	2,325 (60.4)	1,526 (39.6)	
Obese (BMI ≥30 kg/m^2^)	3,232 (61.8)	1,998 (38.2)	
**Diabetes status**	**7,303 (59.8)**	**4,911 (40.2)**	
No diabetes	3,804 (53.5)	3,303 (46.5)	<.001
Prediabetes	1,689 (62.2)	1,026 (37.8)	
Type 2 diabetes	1,810 (75.7)	582 (24.3)	
**Hypertension**	**7,303 (59.8)**	**4,911 (40.2)**	
No	3,628 (53.1)	3,200 (46.9)	<.001
Yes	3,675 (68.2)	1,711 (31.8)	
**Hyperlipidemia**	**7,303 (59.8)**	**4,911 (40.2)**	
No	4,508 (55.0)	3,685 (45.0)	<.001
Yes	2,795 (69.5)	1,226 (30.5)	

Abbreviations: BMI, body mass index; CI, confidence interval; PCP, primary care provider.

a Sugar-sweetened beverage includes sugar-sweetened soda, sweetened ice tea, sports drinks, and other fruit-flavored drinks but does not include 100% juice. One serving equals 12 ounces.

b Percentages may not total 100% because of rounding.

c χ^2^ test was used as the test statistic.

d One serving equals 1 medium apple, a handful of broccoli, or a cup of carrots.

e Participating in any physical activities other than one’s regular job or exercises such as running, calisthenics, golf, gardening, or walking for exercise in past 30 days.

f Walking or biking more than 10 blocks as part of getting to and from work, school, public transportation or to do errands in past 30 days.

 As measured by crude odds ratios with 90% CIs in the univariable model, all covariates except for race/ethnicity and exercising within the last 30 days were significantly associated with daily SSB intake (Table 3). The multivariable logistic regression model demonstrated that patients who drank ≥1 servings of a SSB daily were more likely to be aged 18 to 29 years versus age ≥70 years (adjusted OR = 1.55, 95% CI = 1.24–1.93), a current smoker versus never smoking (adjusted OR = 1.99, 95% CI = 1.78–2.22), never eating fruits and/or vegetables daily compared with eating ≥5 servings daily (adjusted OR = 2.61, 95% CI = 1.97–3.45), walking or biking ≤10 blocks daily in the last 30 days (adjusted OR = 1.26, 95% CI = 1.15–1.38). Furthermore, patients who drank ≥1 servings of a SSB daily were less likely to be preferentially speaking Spanish versus English (adjusted OR = 0.85, 95% CI = 0.74–0.97), female versus male (adjusted OR = 0.82, 95% CI = 0.74–0.91), diagnosed with type 2 diabetes versus not having diabetes (adjusted OR = 0.56, 95% CI = 0.47–0.65) and diagnosed with hypertension versus no hypertension (adjusted OR = 0.86, 95% CI = 0.76–0.97).

**Table 3:** Crude and Adjusted Odds Ratio of Consuming ≥1 Servings of Sugar-Sweetened Beverages Daily by Characteristics Using Logistic Regression, Among Adult Patients Seen at Federally Qualified Health Centers in Bronx, New York, 2013

Variable	≥ 1 Serving/Day of Sugar-Sweetened Beverage^a^	
	Crude OR (90% CI)	Adjusted OR^b^ (95% CI)
**Demographic variables**		
**Age (years)**		
18–29	2.93 (2.61–3.30)	1.55 (1.24–1.93)
30–39	1.99 (1.76–2.25)	1.08 (0.87–1.35)
40–49	1.65 (1.46–1.86)	0.99 (0.81–1.21)
50–59	1.24 (1.10–1.40)	0.83 (0.69–1.01)
60–69	1.02 (0.90–1.17)	0.86 (0.71–1.05)
≥ 70	1 [Reference]	1 [Reference]
**Sex**		
Male	1 [Reference]	1 [Reference]
Female	0.82 (0.77–0.88)	0.82 (0.74–0.91)
**Race/ethnicity**		
White	1 [Reference]	1 [Reference]
Black or African American	0.77 (0.50 −1.21)	0.76 (0.39–1.49)
Hispanic or Latino	0.78 (0.51–1.21)	0.78 (0.40–1.51)
Asian	0.46 (0.24–0.88)	0.61 (0.24–1.53)
Other	0.78 (0.47–1.30)	0.73 (0.34–1.56)
Unknown/missing/refused	0.76 (0.49–1.18)	0.78 (0.40–1.51)
**Preferred language**		
English	1 [Reference]	1 [Reference]
Spanish	0.71 (0.66–0.76)	0.85 (0.74–0.97)
Vietnamese	0.43 (0.24–0.73)	0.67 (0.32–1.31)
Cambodian	0.36 (0.19–0.65)	0.63 (0.25–1.41)
Other	0.56 (0.42 −0.72)	0.68 (0.47–0.97)
**Health behaviors**		
**Smoking status**		
Never smoked	1.00 [Reference]	1.00 [Reference]
Former smoker	1.04 (0.96–1.13)	NA **c**
Current smoker	2.06 (1.90–2.23)	1.99 (1.78–2.22)
**Fruit and/or vegetable consumption (servings/d)^d^**		
None	2.93(2.42–3.57)	2.61 (1.97–3.45)
1-4 servings	1.84 (1.52–2.23)	1.85 (1.42–2.44)
5 or more servings	1 [Reference]	1 [Reference]
**Exercise^e^**		
Yes	1.00 [Reference]	NA **c**
No	1.02 (0.95–1.08)	NA **c**
**Walk/Bike^f^**		
Yes	1 [Reference]	1 [Reference]
No	1.23 (1.15–1.30)	1.26 (1.15–1.38)
**Health services**		
**Insurance**		
Medicare or commercial	1 [Reference]	1 [Reference]
Medicaid or uninsured	1.47 (1.38–1.57)	1.09 (0.98–1.20)
**Outpatient PCP visits per year**		
1	1 [Reference]	1 [Reference]
2–6	0.81 (0.73–0.88)	0.94 (0.81–1.09)
7–12	0.64 (0.58–0.71)	0.94 (0.80–1.10)
≥13	0.59 (0.53–0.66)	0.91 (0.77–1.07)
**Health conditions**		
**Weight status**		
Underweight/normal weight (BMI <25 kg/m^2^)	1 [Reference]	1 [Reference]
Overweight (BMI 25–29.99 kg/m^2^)	0.82 (0.75–0.89)	0.99 (0.87–1.11)
Obese (BMI ≥30 kg/m^2^)	0.77 (0.71–0.83)	1.02 (0.91–1.15)
**Diabetes status**		
No diabetes	1 [Reference]	1 [Reference]
Prediabetes	0.70 (0.65–0.75)	0.91 (0.81–1.02)
Type 2 diabetes	0.37 (0.34–0.40)	0.56 (0.47–0.65)
**Hypertension**		
No	1 [Reference]	1 [Reference]
Yes	0.53 (0.50–0.56)	0.86 (0.76–0.97)
**Hyperlipidemia**		
No	1 [Reference]	1 [Reference]
Yes	0.54 (0.50–0.57)	0.96 (0.85–1.09)

Abbreviations: BMI, body mass index; CI, confidence interval; NA, not applicable; OR, odds ratio; PCP, primary care provider.

a Sugar-sweetened beverage includes sugar-sweetened soda, sweetened ice tea, sports drinks, and other fruit-flavored drinks but does not include 100% juice. One serving equals 12 ounces.

b The logistic model was adjusted for age, preferred language, race/ethnicity, sex, smoking status, daily servings of fruit and/or vegetable consumption, walk/bike, health insurance, number of yearly PCP outpatient visits, BMI, diabetes status, hypertension and hyperlipidemia.

c Not applicable because the variable was not significant in the unadjusted model and therefore was not included in the adjusted model.

d One serving equals 1 medium apple, a handful of broccoli, or a cup of carrots.

e Participating in any physical activities other than one’s regular job or exercises such as running, calisthenics, golf, gardening, or walking for exercise in past 30 days.

f Walking or biking more than 10 blocks as part of getting to and from work, school, public transportation or to do errands in past 30 days.

## Discussion

To our knowledge, this is the first study examining the relationship of SSB consumption with demographic, health behavior, health service, and health characteristics among adult patients in a clinical setting by using standardized health behavior questions integrated into the EHR system. Our analysis demonstrates that SSB intake may be interrelated with other unhealthy behaviors, associated with younger age, and inversely associated with individuals who have diagnosed type 2 diabetes and hypertension.

Our findings are akin to previous studies in that SSB intake is highly associated with younger age (1,13). Previous studies have mixed results regarding the association of health behaviors and SSB consumption (13–15). The discrepancies may be caused by many factors, such as the use of various definitions of the health behaviors in different studies (27).

This study also found that a known diagnosis of type 2 diabetes is statistically associated with a reduced likelihood of daily SSB consumption. Although the causal relationship between SSB intake and diagnosis of type 2 diabetes cannot be established because of the cross-sectional study design, it does suggest that patients decreased their sugar consumption as a reactive measure to their diagnosis of type 2 diabetes.

Methods used in our study to integrate health behavior RAQs into the EHR system of FQHCs can provide opportunities for both primary care centers and public health agencies to improve the health of patients and populations respectively. Programming the EHR to automatically prompt nurses to ask patients the RAQs annually and allowing providers the ability to review the answers to the RAQs offer clinicians a unique opportunity to discuss lifestyle changes with their patients. Patients are more likely to attempt behavior changes when counseled to do so by a health care provider (28,29). Given the findings of our study, SSB intake may be a useful indicator of other unhealthy behaviors and could aid clinicians in identifying and counseling patients. Having a record of a patient’s health behaviors can allow the provider to assess behavior changes over time. Furthermore, primary care centers can use this information at an aggregate level to assess what unhealthy behaviors are prevalent among their patient population compared with the community population and implement health center initiatives to address those behaviors (19). This was the primary reason why the FQHC agreed to incorporate the RAQs into the health centers’ workflow. Primary care centers partnering with public health entities can coordinate and promote use of public services related to specific health behaviors.

Furthermore, studies using methods similar to ours can reveal potential mechanisms underlying the relationship between community factors (eg, food environment), health behaviors (eg, SSB consumption), and health outcomes (eg, obesity). Most current epidemiological approaches to health research reported these variables at an aggregate level by various data sources. However, the mechanisms that relate these variables cannot be ascertained with only aggregate-level data. Future studies using patient-level data, obtained using methods outlined in our study, in conjunction with community factor data provided by public health agencies, may further our understanding of these mechanisms.

This study has several limitations. First, all health behavior covariates are self-reported and may be subject to recall bias. Second, the daily fruit and vegetable consumption RAQ does not define what is a fruit or a vegetable. Some participants might have included ketchup or potatoes in their daily fruit and vegetable tally, and others may have difficulty estimating the amount of fruits or vegetables that were incorporated into a mixed dish, stew, or soup. Finally, 39% of participants in the study had unknown, unanswered, or declined data regarding their race/ethnicity. The likely reason is that many participants’ racial backgrounds are diverse and could not be categorized into 1 of the racial groups listed in the questionnaire. Future studies may benefit from giving more racial options in the questionnaire.

More than 40% of adult patients consumed 1 or more SSBs daily. Given the distinct characteristic associations and predictors of SSB consumption, our findings could aid clinicians in identifying and counseling patients about unhealthy behaviors. Future studies should explore how patient-level health behavior data captured in EHRs can be used to improve the health of patients and communities.

## Appendix. Crude Population Characteristics of Adults Aged 18 or Older in Neighborhoods Surrounding the Federally Qualified Health Centers, 2012

**Table:**

Variable	Fordham-Bronx Park Neighborhood^a^	South Bronx Neighborhood^b^	Northeast Bronx Neighborhood^c^

	n (%^d^)	n (%^d^)	n (%^d^)
**Demographic variables**			
**Age, y** ^e^			
18–29	53,736 (29.2)	110,011 (28.7)	31,237 (21.4)
30–39	36,030 (19.6)	74,342 (19.4)	23,206 (15.9)
40–49	34,686 (18.9)	75, 613 (19.7)	27,997 (19.2)
50–59	28,229 (15.4)	58,968 (15.4)	25,351 (17.4)
60–69	16,588 (9.0)	36,214 (9.4)	18,625 (12.8)
≥70	14,573 (7.9)	28,760 (7.5)	19,632 (13.4)
**Sex** ^e^			
Male	84,652 (46.1)	172,683 (45.0)	62,528 (42.7)
Female	99,190 (54.0)	211,225 (55.2)	83,520 (57.2)
**Race/ethnicity** ^e^			
White	18,865 (10.3)	6,622 (1.7)	18,712 (12.8)
Black or African American	46,612 (25.4)	117,572 (30.6)	85,889 (58.8)
Hispanic or Latino	105,092 (57.2)	249, 815 (65.1)	33,302 (22.8)
Asian	9,768 (5.3)	4,673 (1.2)	4,126 (2.8)
Other	3,505 (1.9)	5,226 (1.4)	4,019 (2.8)

^a^ United Health Fund neighborhood encompassing zip codes 10458, 10467, 10468; Family Health Center is located in this neighborhood.

^b^ United Health Fund neighborhood encompassing zip codes 10451, 10452, 10453, 10454, 10455, 10456, 10457, 10459, 10460, 10474; West Farms Family Practice and Comprehensive Health Care Center are in this neighborhood.

^c^ United Health Fund neighborhood encompassing zip codes 10466, 10469, 10470,10475; Williamsbridge Family Practice is in this neighborhood.

^d^ Percentages may not total 100% because of rounding.

^e^ US Census Bureau. American FactFinder. 2010 Demographic profile data. http://factfinder2.census.gov. Accessed October 8, 2014.

## References

[1] Ogden CL , Kit BK , Carroll MD , Park S . Consumption of sugar drinks in the United States, 2005–2008. NCHS data brief 2011 (71):1–8. 22617020

[2] Hu FB . Resolved: there is sufficient scientific evidence that decreasing sugar-sweetened beverage consumption will reduce the prevalence of obesity and obesity-related diseases. Obes Rev 2013;14(8):606–19. 10.1111/obr.12040 23763695 PMC5325726

[3] Sources of calories from added sugars among the US population, 2005–06. Applied Research Program Web site. National Cancer Institute. http://appliedresearch.cancer.gov/diet/foodsources/added_sugars/. Accessed April 29, 2014.

[4] Dhingra R , Sullivan L , Jacques PF , Wang TJ , Fox CS , Meigs JB , Soft drink consumption and risk of developing cardiometabolic risk factors and the metabolic syndrome in middle-aged adults in the community. Circulation 2007;116(5):480–8. 10.1161/CIRCULATIONAHA.107.689935 17646581

[5] Malik AH , Akram Y , Shetty S , Malik SS , Yanchou Njike V . Impact of sugar-sweetened beverages on blood pressure. Am J Cardiol 2014;113(9):1574–80. 10.1016/j.amjcard.2014.01.437 24630785

[6] Malik VS , Pan A , Willett WC , Hu FB . Sugar-sweetened beverages and weight gain in children and adults: a systematic review and meta-analysis. Am J Clin Nutr 2013;98(4):1084–102. 10.3945/ajcn.113.058362 23966427 PMC3778861

[7] Malik VS , Popkin BM , Bray GA , Després J-P , Willett WC , Hu FB . Sugar-sweetened beverages and risk of metabolic syndrome and type 2 diabetes: a meta-analysis. Diabetes Care 2010;33(11):2477–83. 10.2337/dc10-1079 20693348 PMC2963518

[8] Mattes RD , Shikany JM , Kaiser KA , Allison DB . Nutritively sweetened beverage consumption and body weight: a systematic review and meta-analysis of randomized experiments. Obes Rev 2011;12(5):346–65. 10.1111/j.1467-789X.2010.00755.x 20524996 PMC3169649

[9] Yoon PW , Batian B , Anderson RN , Collins JL , Jaffe H . Potentially preventable deaths from the five leading causes of death — United States, 2008–2010. MMWR Morb Mortal Wkly Rep 2014;63(17):369–74. 24785982 PMC4584887

[10] Drewnowski A , Specter SE . Poverty and obesity: the role of energy density and energy costs. Am J Clin Nutr 2004;79(1):6–16. 14684391 10.1093/ajcn/79.1.6

[11] Kit BK , Fakhouri TH , Park S , Nielsen SJ , Ogden CL . Trends in sugar-sweetened beverage consumption among youth and adults in the United States: 1999–2010. Am J Clin Nutr 2013;98(1):180–8. 10.3945/ajcn.112.057943 23676424 PMC10401613

[12] Mozaffarian D , Hao T , Rimm EB , Willett WC , Hu FB . Changes in diet and lifestyle and long-term weight gain in women and men. N Engl J Med 2011;364(25):2392–404. 10.1056/NEJMoa1014296 21696306 PMC3151731

[13] Park S , Pan L , Sherry B , Blanck HM . Consumption of sugar-sweetened beverages among US adults in 6 states: Behavioral Risk Factor Surveillance System, 2011. Prev Chronic Dis 2014;11:E65. http://www.cdc.gov/pcd/issues/2014/13_0304.htm. Accessed May 9, 2014. 10.5888/pcd11.130304 24762529 PMC4008944

[14] Rehm CD , Matte TD , Van Wye G , Young C , Frieden TR . Demographic and behavioral factors associated with daily sugar-sweetened soda consumption in New York City adults. J Urban Health 2008;85(3):375–85. 10.1007/s11524-008-9269-8 18347992 PMC2329746

[15] Sharkey JR , Johnson CM , Dean WR . Less-healthy eating behaviors have a greater association with a high level of sugar-sweetened beverage consumption among rural adults than among urban adults. Food Nutr Res 2011;55. 21845142 10.3402/fnr.v55i0.5819 PMC3153312

[16] LeBlanc ES , O’Connor E , Whitlock EP , Patnode CD , Kapka T . Effectiveness of primary care-relevant treatments for obesity in adults: a systematic evidence review for the US Preventive Services Task Force. Ann Intern Med 2011;155(7):434–47. 10.7326/0003-4819-155-7-201110040-00006 21969342

[17] Kukafka R , Ancker JS , Chan C , Chelico J , Khan S , Mortoti S , Redesigning electronic health record systems to support public health. J Biomed Inform 2007;40(4):398–409. 10.1016/j.jbi.2007.07.001 17632039

[18] Glasgow RE , Kaplan RM , Ockene JK , Fisher EB , Emmons KM . Patient-reported measures of psychosocial issues and health behavior should be added to electronic health records. Health Aff (Millwood) 2012;31(3):497–504. 10.1377/hlthaff.2010.1295 22392660

[19] Chambers EC , Wong BC , Riley RW , Hollingsworth N , Blank AE , Myers C , Combining clinical and population-level data to understand the health of neighborhoods. Am J Public Health 2014. Forthcoming. 10.2105/AJPH.2014.302326 PMC4323686 25602860

[20] Bedell JF , Hollingsworth N , Larrier E , Beckman A , Candalla B , Taylor DO , Bronx-CATCH: a multi-stakeholder collaboration to improve community health in the Bronx, NY. Poster session presented at the American Public Health Association 140th Annual Meeting & Expo 2012 Oct 27–31; San Francisco, CA.

[21] Behavioral Risk Factor Surveillance System. Atlanta (GA): US Department of Health and Human Services, Centers for Disease Control and Prevention. http://www.cdc.gov/brfss/. Accessed January 22, 2014.

[22] New York City Department of Health and Mental Hygiene. Epiquery: NYC Interactive Health Data System-[Community Health Survey 2012]. http://nyc.gov/health/epiquery. Accessed December 3, 2013.

[23] Executive summary: Standards of medical care in diabetes — 2010. Diabetes Care 2010;33(Suppl 1):S4–10. 10.2337/dc10-S004 20042774 PMC2797389

[24] Klompas M , Eggleston E , McVetta J , Lazarus R , Li L , Platt R . Automated detection and classification of type 1 versus type 2 diabetes using electronic health record data. Diabetes Care 2013;36(4):914–21. 10.2337/dc12-0964 23193215 PMC3609529

[25] Hirsch AG , Scheck McAlearney A . Measuring diabetes care performance using electronic health record data: the impact of diabetes definitions on performance measure outcomes. Am J Med Qual 2013;29(4):292–99. 24006028 10.1177/1062860613500808

[26] American Diabetes Association. Standards of medical care in diabetes — 2014. Diabetes Care 2014;37 (Suppl 1):S14–80. 10.2337/dc14-S014 24357209

[27] Ebrahim S , Montaner D , Lawlor DA . Clustering of risk factors and social class in childhood and adulthood in British women’s heart and health study: cross sectional analysis. BMJ 2004;328(7444):861. 10.1136/bmj.38034.702836.55 15006898 PMC387475

[28] Thande NK , Hurstak EE , Sciacca RE , Giardina EG . Management of obesity: a challenge for medical training and practice. Obesity (Silver Spring) 2009;17(1):107–13. 10.1038/oby.2008.478 19107125

[29] Rose SA , Poynter PS , Anderson JW , Noar SM , Conigliaro J . Physician weight loss advice and patient weight loss behavior change: a literature review and meta-analysis of survey data. Int J Obes (Lond) 2013;37(1):118–28. 22450855 10.1038/ijo.2012.24

